# Can neonicotinoid and pyrrole insecticides manage malaria vector resistance in high pyrethroid resistance areas in Côte d'Ivoire?

**DOI:** 10.1186/s12936-024-04917-y

**Published:** 2024-05-22

**Authors:** Armand K. Ekra, Constant A. V. Edi, Guy Constant N. Gbalegba, Julien Z. B. Zahouli, Mathias Danho, Benjamin G. Koudou

**Affiliations:** 1 UMRI 28, Sciences Agronomiques et Procédés de Transformation, Laboratoire de Zoologie Agricole et Entomologie, Institut National Polytechnique Félix Houphouët-Boigny (INP-HB), Yamoussoukro, République de Côte d’Ivoire; 2 Centre Suisse de Recherches Scientifiques en Côte d’Ivoire, Abidjan, République de Côte d’Ivoire; 3 Programme National de Lutte Contre le Paludisme, Abidjan, République de Côte d’Ivoire; 4 Centre d’Entomologie Médicale et Vétérinaire, Université Alassane Ouattara, Bouaké, République de Côte d’Ivoire; 5 Laboratoire d’Entomologie, UFR Sciences de la Nature, Université Nangui Abrogoua, Abidjan, République de Côte d’Ivoire

**Keywords:** Insecticide resistance, *Anopheles gambiae*, Clothianidin, Chlorfenapyr, Malaria, Côte d’Ivoire

## Abstract

**Background:**

*Anopheles* mosquito resistance to insecticide remains a serious threat to malaria vector control affecting several sub-Sahara African countries, including Côte d’Ivoire, where high pyrethroid, carbamate and organophosphate resistance have been reported. Since 2017, new insecticides, namely neonicotinoids (e.g.; clothianidin) and pyrroles (e.g.; chlorfenapyr) have been pre-qualified by the World Health Organization (WHO) for use in public health to manage insecticide resistance for disease vector control.

**Methods:**

Clothianidin and chlorfenapyr were tested against the field-collected *Anopheles gambiae* populations from Gagnoa, Daloa and Abengourou using the WHO standard insecticide susceptibility biossays. *Anopheles gambiae* larvae were collected from several larval habitats, pooled and reared to adulthood in each site in July 2020. Non-blood-fed adult female mosquitoes aged 2 to 5 days were exposed to diagnostic concentration deltamethrin, permethrin, alpha-cypermethrin, bendiocarb, and pirimiphos-methyl. Clothianidin 2% treated papers were locally made and tested using WHO tube bioassay while chlorfenapyr (100 µg/bottle) was evaluated using WHO bottle assays. Furthermore, subsamples of exposed mosquitoes were identified to species and genotyped for insecticide resistance markers including the knock-down resistance (*kdr*) west and east, and acetylcholinesterase (*Ace*-1) using molecular techniques.

**Results:**

High pyrethroid resistance was recorded with diagnostic dose in Abengourou (1.1 to 3.4% mortality), in Daloa (15.5 to 33.8%) and in Gagnoa (10.3 to 41.6%). With bendiocarb, mortality rates ranged from 49.5 to 62.3%. Complete mortality (100% mortality) was recorded with clothianidin in Gagnoa, 94.9% in Daloa and 96.6% in Abengourou, while susceptibility (mortality > 98%) to chlorfenapyr 100 µg/bottle was recorded at all sites and to pirimiphos-methyl in Gagnoa and Abengourou. *Kdr-west* mutation was present at high frequency (0.58 to 0.73) in the three sites and *Kdr*-*east* mutation frequency was recorded at a very low frequency of 0.02 in both Abengourou and Daloa samples and absent in Gagnoa. The *Ace*-1 mutation was present at frequencies between 0.19 and 0.29 in these areas. *Anopheles coluzzii* represented 100% of mosquitoes collected in Daloa and Gagnoa, and 72% in Abengourou.

**Conclusions:**

This study showed that clothianidin and chlorfenapyr insecticides induce high mortality in the natural and pyrethroid-resistant *An. gambiae* populations in Côte d’Ivoire. These results could support a resistance management plan by proposing an insecticide rotation strategy for vector control interventions.

## Background

Worldwide, the number of malaria cases is estimated at 247 million in 2021 in 84 malaria-endemic countries (including French Guyana), up from 245 million in 2020 [1]. *Plasmodium falciparum* is the most deadly infective parasite in the world. The greater virulence of *P. falciparum* compared to other human malaria parasites is attributable to the ability of parasites to infect red blood cell stages of different ages, thereby contributing to a higher parasite biomass, and the unique capability of *P. falciparum*-infected erythrocytes (IEs) to sequester in the microcirculation [2]. In 2021, the WHO African Region will account for around 95% (234 million) of the world's estimated cases. In the WHO African Region, malaria incidence fell from 373 to 225 cases per 1000 population at risk of malaria over the period 2000–2019 before rising again to 234 in 2020, mainly due to the disruption of services during the COVID-19 pandemic. By 2021, the incidence of malaria has fallen to 229 cases per 1,000 population [1]. Malaria kills four people every day in Côte d'Ivoire, including three children under the age of five. The number of deaths due to malaria fell from 3222 in 2017 to 1316 in 2020. In other words, the mortality rate has fallen by around 50%. Despite this reduction, malaria remains a major public health challenge, as it is still the leading cause of consultations in the country's health facilities [3].

Malaria vector resistance to insecticide represents a serious threat to vector control [4–6]. Insecticide resistance of malaria vectors to different insecticides and insecticide classes was reported in many countries around the world and particularly in Côte d'Ivoire [7–9]. Over the years, resistance of malaria vectors to insecticides was attributed to the intensive distribution and use of insecticide-treated nets (ITNs) and indoors residual spraying (IRS) [10]. In addition, the abusive and uncontrolled use of insecticides through various agricultural practices have been reported as contributors to the selection of resistance in malaria vectors [11–13]. Though pyrethroids represent the mainly class of insecticide currently used for the impregnation of long-lasting insecticidal nets (LLINs), a widespread resistance of malaria vectors to pyrethroids is unfortunately reported in most of the endemic countries [14–17]. The emergence of resistance to carbamates and organophosphates is becoming increasingly widespread [18]. In addition, resistance to other classes of insecticides like organochlorines was also reported in several West African countries [9, 15, 19–21]. Thus, the resistance of malaria vectors to all commonly used classes of insecticide in public health is compromising malaria control efforts in many countries requiring a good insecticide resistance management plan [22, 23]. Because of the resistance of vectors to insecticides and the heterogeneity of malaria endemicity in Côte d'Ivoire, the National Malaria Control Programme (NMCP) had stratified the distribution of LLINs in 2021. For instance, during the last mass distribution campaign in April 2021, LLINs were distributed in 113 districts across the country [24]. Standard LLINs were distributed in 84 districts; PBO-containing LLINs were distributed in 11 districts, and 18 health districts received Interceptor® G2 (IG2) LLINs (unpublished data). The national take-up rate is around 63%. This gap is due to the poor results achieved in major cities, such as Abidjan, where the rate fluctuates between 23 and 43% [24]. Despite all these efforts, the entomological data relating to the resistance of malaria vectors remains worrying [21].

Therefore, finding new, highly effective molecules could enable malaria-endemic countries to effectively control the vectors. New insecticides of the neonicotinoid and pyrrole classes have recently been prequalified by the WHO for use in the public health vector control through IRS and ITNs [25, 26]. Chlorfenapyr is a pyrrole insecticide commonly used against mites and termites, which functions as an oxidative phosphorylation uncoupler. This compound disrupts the proton gradient across mitochondrial membranes, interrupting ATP synthesis and ultimately resulting in death of the organism [27, 28]. Neonicotinoids are the most widely sold and used class of insecticides in the world and have been used in agriculture since their introduction in the 1990s. Seed dressing and soil treatment applications account for about 60% of neonicotinoid use [29]. Studies with some of the molecules in these chemical families have shown good results at specific concentrations [17, 30, 31]. Furthermore, recent research studies have demonstrated the malaria impact of chlorfenapyr in combination with a pyrethroid insecticide-based ITNs [32]. Clothianidin-based products applied as IRS insecticides showed good results in several locations [33, 34].

Following the increasing resistance of malaria vectors to insecticide in the country [35] compromising vector control efforts, an insecticide resistance monitoring was conducted in Côte d'Ivoire to evaluate the susceptibility of the vectors to clothianidin (neonicotinoid) and chlorfenapyr (pyrrole) for better resistance management. The present study presents the susceptibility status of wild strains of *Anopheles gambiae *sensu lato (*s.l*.) in three agricultural areas in Côte d’Ivoire.

## Methods

### Study area

**Fig. 1:**
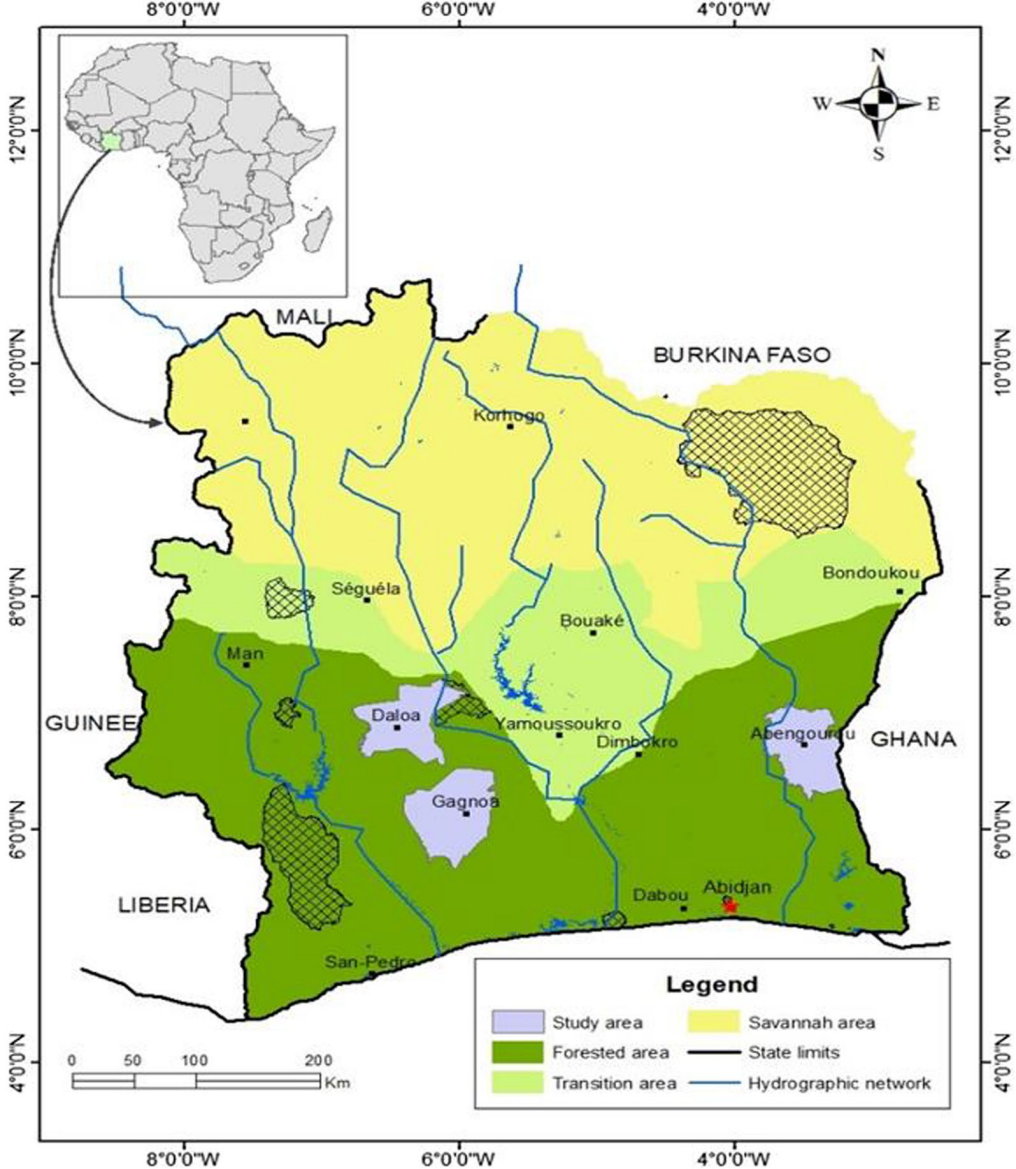
Map showing the study sites in Côte d’Ivoire

The study was conducted in three districts of Côte d'Ivoire, Abengourou in the East and Daloa and Gagnoa in the Central-West. The three localities had in common the urban agricultural activities within the city including rice and vegetable cultivation. The sites have suitable malaria vector larval habitats. The populations of Daloa and Gagnoa received Interceptor® G2 (IG2) mosquito nets during the latest mass distributions of LLINs by the NMCP of Côte d’Ivoire. In Abengourou, the mosquito nets distributed to protect people from mosquitoes were LLINs containing PBO. Abengourou (6° 43′ 47″ north, 3° 29′ 47″ west) is at about 210 km from Abidjan, close to the border of Ghana with an average annual temperature of 27 °C and relative humidity (RH) of 82%. Abengourou is part of the old cocoa loop and is one of the reservoirs of the Ivorian food industry. Daloa (6° 27′ 00″ north, 5° 56′ 00″ west) is at about 383 km from Abidjan, with a mean annual temperature of 27 °C and RH of 86%. Gagnoa (6° 08′ 00″ north, 5° 56′ 00″ west) is at about 285 km from Abidjan (Fig. 1) with an average annual temperature of 26 °C and a RH of 90%.

### Mosquito larvae collection

Mosquito larvae were collected in the different study areas using the dipping method as described by Service in 1993 [36]. Larvae were collected over a two-week period in July 2020. Mosquito larvae were collected in puddles around homes, in rice fields and between the plant rows of market gardens. They were provided access to powdered Tetramin fish food and were reared to adults under laboratory standard conditions (i.e., 25–28 °C temperature, 70–80% RH, and 12:12 light: dark photoperiod) [4]. Adult mosquitoes were maintained with 10% sucrose solution [37, 38].

### WHO diagnostic bioassays

Two to five days non-blood fed adult female mosquitoes were exposed to 0.05% deltamethrin, 0.75% permethrin, 0.05% alpha-cypermethrin, 0.1% bendiocarb, 0.25% pirimiphos-methyl for one hour, using WHO tubes and criteria [4]. In addition to the diagnostic doses, intensity tests were carried out using 5X and 10X doses. Mosquitoes exposed were given access to 10% sucrose solution in WHO holding tubes after exposure time. The knock-down effect was recorded after 60 min of exposure and mortality was recorded after 24 h. For clothianidin, 2.0%-impregnated papers were made locally by diluting 264 mg of clothianidin based SumiShield formulation in 20 ml of distilled water [31]. Two millilitres of the solution were used to impregnate the 12 × 15 cm filter paper. Exposure was also done for one hour and mortality recorded up to seven days post exposure. For each replicate, about fifty mosquitoes were exposed to either silicone oil treated papers (pyrethroids), olive oil (carbamate and organophosphate) or distilled water impregnated papers (clothianidin) as a control in two separate tubes. Exposed mosquitoes were then stored on silica gel until DNA extraction according to dead or living status.

### WHO Bottle bioassay

WHO Bottle bioassays were conducted using chlorfenapyr (100 µg/bottle) based on a literature review and previous studies carried out in Côte d’Ivoire [17, 28, 31, 39]. Each 250 ml bottle and its cap was coated with 1 ml of insecticide solution by rolling and inverting the bottles. In parallel, a control bottle was coated with 1 ml of acetone, following which all bottles were covered with a sheet and left to dry in the dark [31]. For each site, four chlorfenapyr-coated bottles were used and about 20–25 *An. gambiae* females aged 2–5 days were transferred in each bottle and exposed for 60 min. The knock-down effect was recorded after 60 min of exposure, with mortality recorded up to 72 h after exposure.

### DNA extraction and Genotyping assays

Genomic DNA was extracted according to the LIVAK method [40] from individual *An. gambiae s.l.* mosquitoes from each site. TaqMan assays with two labelled fluorochromes probes FAM and HEX were used to screen for the L995F/S (previously referred to as L1014F/S) *kdr* mutations [7] and, the *ace-1*^*R*^ G280S (previously referred to as Ace1-G119S) mutation. Reactions were performed on the Agilent MX3005P qPCR system (Agilent Technologies). The genotype was determined from the fluorescence profiles and bi-directional scatter plots generated in the MX3005P software. The PCR condition was 95 °C for 10 min (1 cycle) following by 40 cycles of 95 °C at 10 s and 60 °C at 45 s, respectively.

### Data analysis

Following exposure of mosquito populations to insecticides, the resistance status of the population was determined using WHO criteria. When recorded mortalities were between 98 and 100% the population was considered as susceptible. Populations showing mortality between 90 and 98% were possible resistance and populations showing mortality below 90% were rated as resistant [4]. Mortality was corrected using Abbott’s formula when the mortality of the control tubes was above 5% and less than 20%. A Fisher test was used to evaluate the difference in mortality between PBO + pyrethroid and each diagnostic dose of pyrethroid insecticides alone. The Anova and Kruskal Wallis tests were used to compare the differences in mortality between 1X, 5X and 10X doses of each pyrethroid. Allelic frequencies were calculated using the formula ƒ(R) = (2n.RR + n.RS)/2N, where n is the number of mosquitoes of a given genotype, RR represents the homozygote resistance allele, RS represents the heterozygote resistance allele, SS represents the susceptible allele, and N is the total number of mosquitoes tested [9]. Allelic frequencies were analyzed and compared using a Chi-squared test. And a general linear model (GLM) was used to compare mortality induced by chlorfenapyr and clothianidin in the three localities. All statistical analyses were carried out using the R 4.1.2 software (RStudio).

## Results

### Insecticide susceptibility

**Fig. 2:**
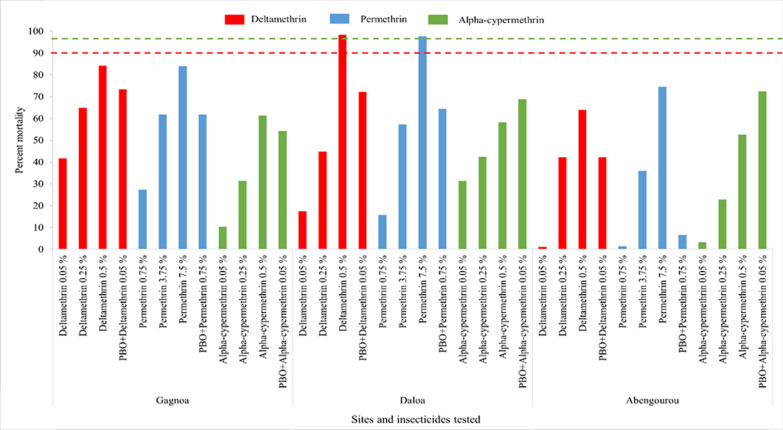
Mortality observed after 24 h with pyrethroid insecticides in *Anopheles gambiae* populations from Côte d’Ivoire in 2020

**Fig. 3:**
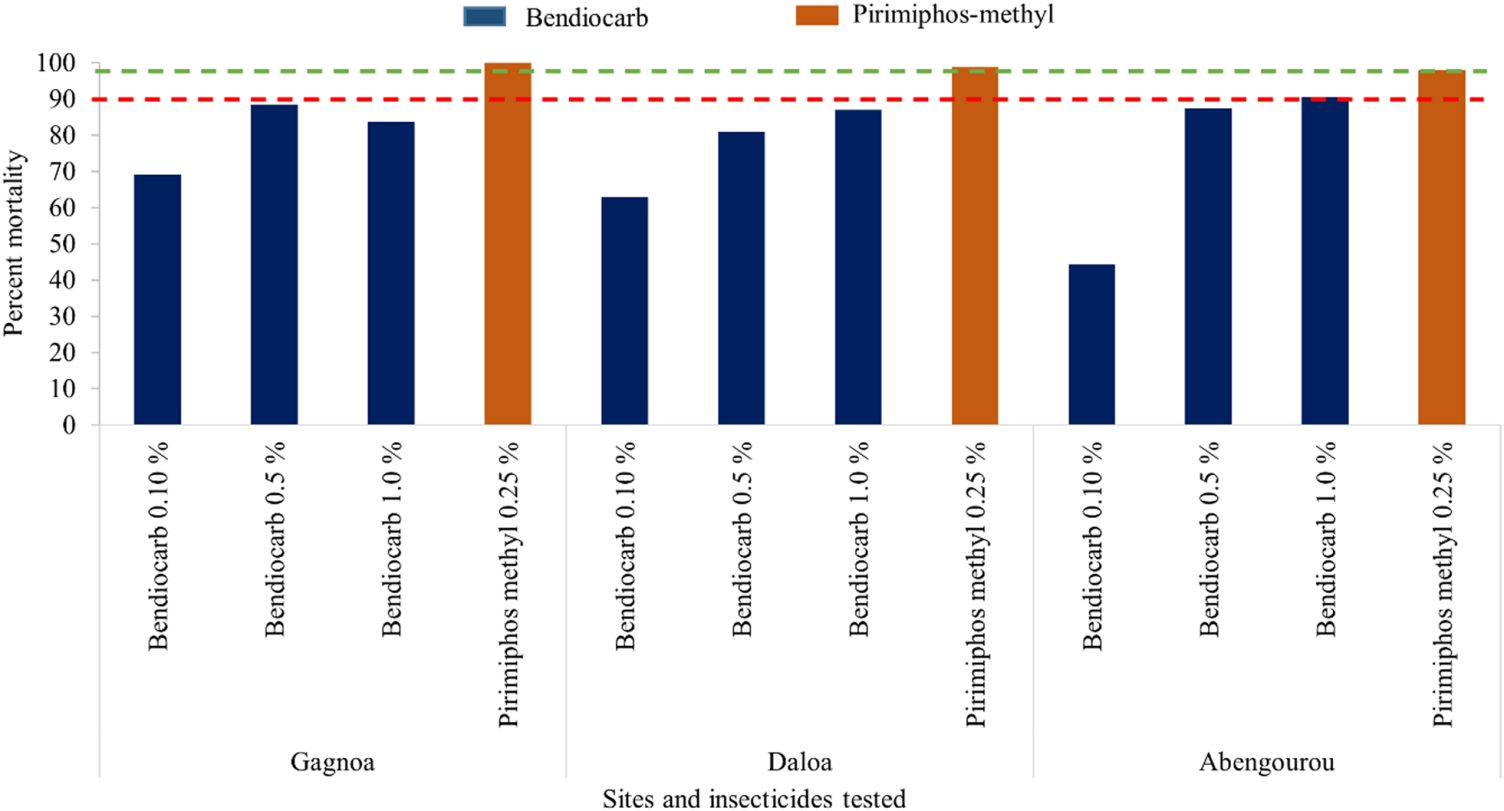
Mortality after 24 h post exposure to bendiocarb and pirimiphos-methyl insecticides in *Anopheles gambiae* populations from Côte d’Ivoire in 2020

A total of 4207 female wild *An. gambiae* (Gagnoa: n = 1199 exposed and n = 81 control; Daloa: n = 1487 exposed and n = 50 control; Abengourou: n = 1307 exposed and n = 83 control) mosquitoes were used to evaluate susceptibility to pyrethroids, pirimiphos-methyl and bendiocarb. Resistance to pyrethroids and bendiocarb was recorded in all three study districts (Fig. 2) while susceptibility was observed for pirimiphos-methyl (Fig. 3). Furthermore, the intensity of the pyrethroid resistance was high in all sites except for permethrin and deltamethrin in Daloa where moderate resistance was recorded. Mortality rates were significantly different according to insecticide concentration (1X, 5X and 10X of pyrethroids) in each site (Gagnoa (deltamethrin: *p* = 0.0003981, permethrin: *p* = 0.0004756, alphacypermethrin: *p* < 0.0001); Abengourou (deltamethrin: *p* = 0.01153, permethrin: *p* = 0.007152, alphacypermethrin: *p* = 0.0004216)) except for alphacypermethrin in Daloa where no significant difference was observed (deltamethrin: *p* = 0.009092, permethrin: *p* < 0.0001, alphacypermethrin: *p* = 0.09402).The resistance to bendiocarb was high at all three sites (Fig. 3). Exposure of *An. gambiae* *s.l.* to PBO before the diagnostic doses of pyrethroids (deltamethrin, permethrin, and alpha-cypermethrin) induced higher mortality compared to the insecticides alone in all study sites (Fig. 2), though the increase in mortality was partial as the resistance status of any of the pyrethroids was not reversed to susceptibility (Fig. 2). There was a significant difference for all insecticides and in all three study sites except for permethrin in Abengourou (Fisher’s Exact test, Gagnoa (deltamethrin: *p* = 0.04994, permethrin: *p* = 0.01131, alphacypermethrin: *p* < 0.0001); Daloa (deltamethrin: *p* = 0.00015, permethrin: *p* < 0.0001, alphacypermethrin: *p* = 0.01319) and Abengourou (deltamethrin: *p* < 0.0001, permethrin: *p* = 0.2788, alphacypermethrin: *p* < 0.0001)).

A total of 657 female wild *An. gambiae* mosquitoes were used to evaluate susceptibility to clothianidine 2% (n = 247 exposed and n = 135 control) and chlorfenapyr 100 µg/bottle (n = 179 exposed and n = 96 control).

**Fig. 4:**
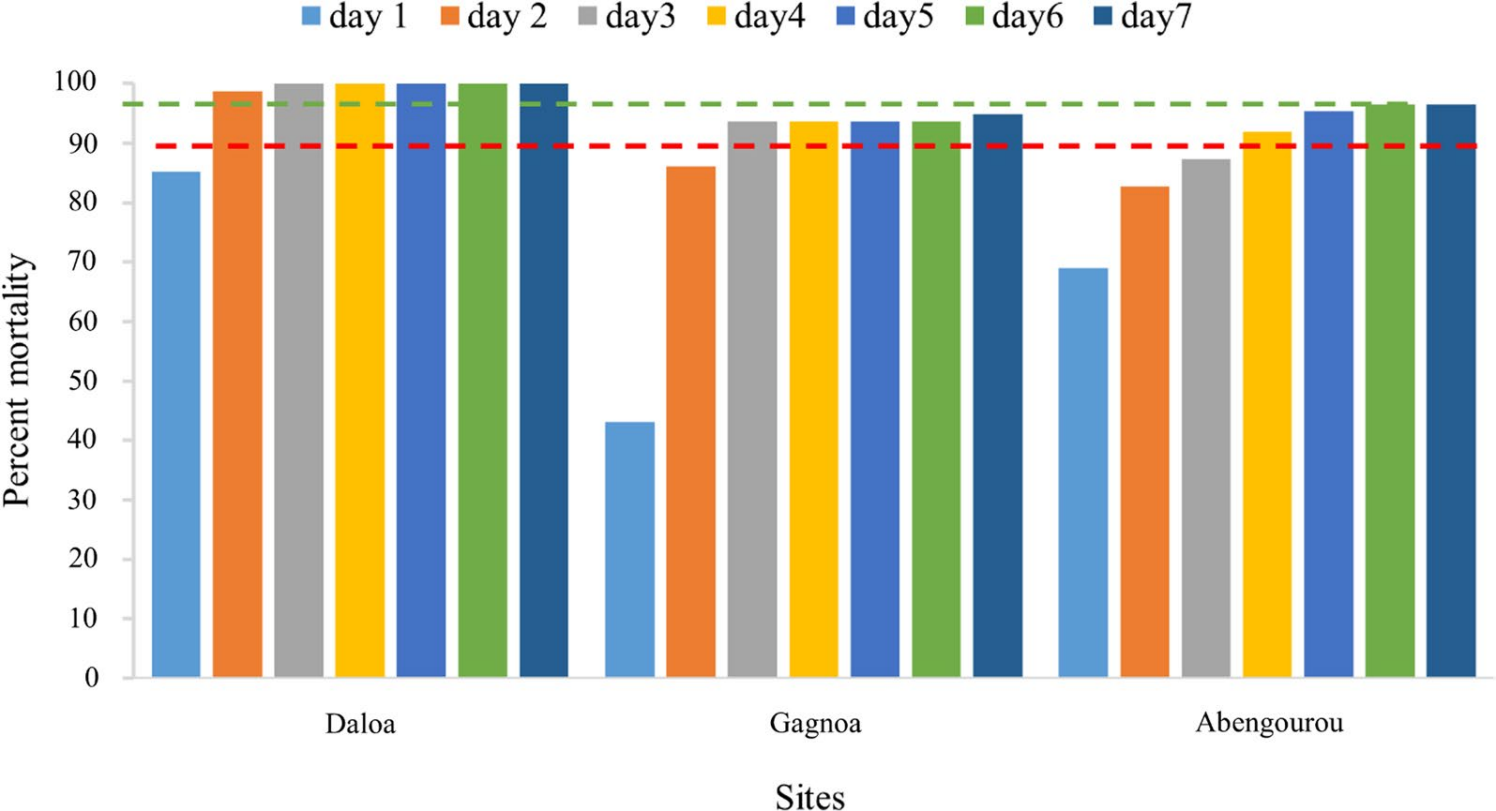
Mortality observed after seven days post exposure to Clothianidin (2%) insecticides in *Anopheles gambiae* populations from Côte d’Ivoire in 2020

**Fig. 5:**
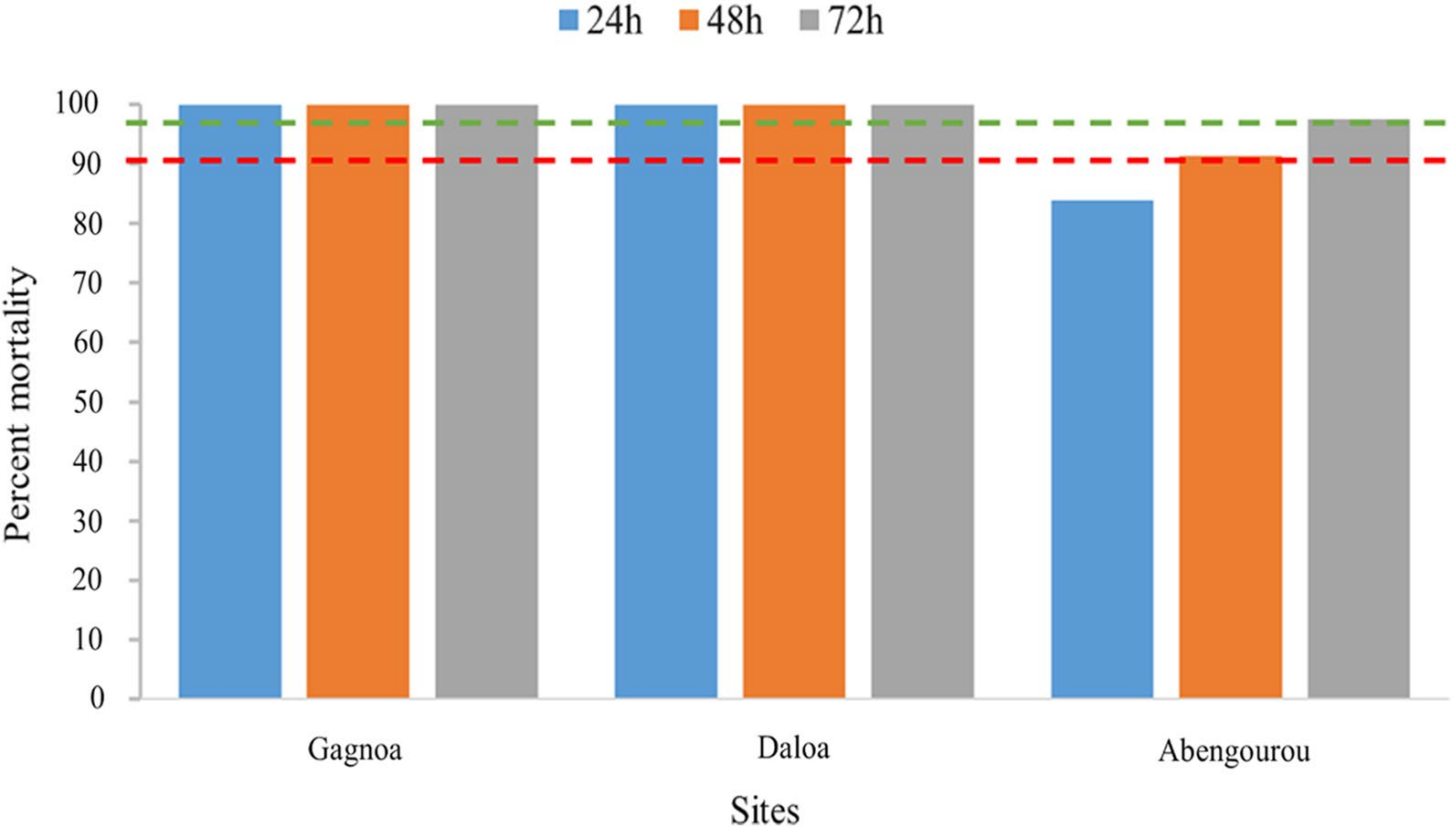
Mortality after 72 h post exposure to chlorfenapyr insecticides in *Anopheles gambiae* populations from Côte d’Ivoire in 2020

With clothianidin 2%, 100% mortality was observed only in Gagnoa. Daloa and Abengourou recorded 94.9% and 96.6% mortality, respectively (Fig. 4) seven days after exposure. There was no significant difference in clothianidin-induced mortality between the three localities (Abengourou – Daloa: p = 0.8645; Abengourou – Gagnoa: p = 0.996; Daloa – Gagnoa: p = 1.0). In contrast, susceptibility to chlorfenapyr 100 µg/bottle was found with more than 98% mortality observed at all of the study sites (Fig. 5). There was no significant difference in mortality induced between these localities (Abengourou – Daloa: p = 0.998; Abengourou – Gagnoa: p = 0.998; Daloa – Gagnoa: p = 1.0).

### Species identification

Table 1 shows the species composition of *An. gambiae s.l*. that was composed of *An. gambiae *sensu stricto (*s.s*)*.* and *Anopheles coluzzii*. *Anopheles gambiae s.l*. in Abengourou were predominantly composed of *An. coluzzii* (72%), followed by 28% *An. gambiae s.s*., while those from Daloa and Gagnoa were 100% *An. coluzzii*.

**Table 1:** Species Identification in the three areas surveyed

District	Total tested	*An. coluzzii*	*An. gambiae* s.s
Abengourou	50	36 (72%)	14 (28%)
Daloa	50	50 (100%)	0 (0%)
Gagnoa	50	50 (100%)	0 (0%)

### Frequencies of *kdr-west*, *kdr-east* and *ace-1* in mosquitoes

A total of 150 mosquitoes were tested to evaluate the frequencies of *kdr*-*west*, *kdr*-*east* and *ace-1*. *kdr*-west (L995F) mutation was present at high frequency (0.58 to 0.73) in the three town. There was no significant difference between the three localities (*χ*^*2*^ = 2.6572, df = 2, *p* = 0.2648). But the *kdr*-east (L995S) mutation was very low in the Abengourou (0.02) and Daloa (0.02) samples and absent in the Gagnoa sample (0.0). And there was no significant difference in the presence of this mutation between the localities (*χ*^*2*^ = 1.1137, df = 2, *p* = 0.57) The *ace*-1 (G280S) mutation was present (0.19 to 0.29) in these areas (Table 2). However there was no significant difference in the presence of this mutation between the localities (*χ*^*2*^ = 1.4214, df = 2, *p* = 0.4913).

**Table 2:** Frequencies of L995F, L995S and G280S in mosquitoes

District	L995F					L995S					G280S				
	Total (n)	RR	RS	SS	Freq	Total (n)	RR	RS	SS	Freq	Total (n)	RR	RS	SS	Freq
Abengourou	24	11	6	7	0.58	23	0	1	22	0.02	21	3	3	15	0.21
Daloa	24	14	7	3	0.73	21	0	1	20	0.02	21	0	8	13	0.19
Gagnoa	25	9	12	4	0.60	24	0	0	24	0	24	0	14	10	0.29

n: number of mosquitoes of a given genotype; Freq: Frequency of resistant alleles; RR: Homozygous with two resistant alleles; RS: Heterozygous with one resistant allele and one susceptible allele; SS: Homozygous with two susceptible alleles

## Discussion

The main objective of this study was to teste the bio-efficacy of clothianidin and chlorfenapyr against natural pyrethroid-resistant populations of *An. gambiae* in Côte d’Ivoire. High pyrethroid resistance was recorded at all study sites. Complete mortality was recorded with clothianidin in Gagnoa, 94.9% in Daloa and 96.6% in Abengourou, while susceptibility to chlorfenapyr 100 µg/bottle was recorded at all sites and to pirimiphos-methyl in Gagnoa and Abengourou.

Resistance of *An. gambiae s.l.* to pyrethroids was observed in the three investigated sites as observed in the whole country over several years [8, 15, 41, 42]. Similarly, several sub-region of countries, such as Burkina Faso and Mali, have also reported cases of resistance [22, 43, 44]. In addition, high resistance intensity was observed to 5X and 10X the diagnostic doses of pyrethroid insecticides. This intensity of resistance could be related to the rapid scale up of pyrethroid-based ITNs for malaria vector control in Côte d’Ivoire, since 2014 [10]. Given the high resistance of vectors to pyrethroids, the tools treated with pyrethroids will be threaten by the resiatnce. Only the 10X-diagnostic doses of deltamethrin and permethrin induced more than 90% mortality in mosquito population from Daloa. These results are really worrying and emphasize the need to find other or alternatives molecules for a better management of malaria vector resistance in Côte d'Ivoire. *Anopheles gambiae* s.l. was also highly resistant to Bendiocarb at all sites showing the limited option of insecticide choices and classes in the country similar to trends observed in several African countries [9, 15, 43, 45]. However, pirimiphos-methyl was still recording susceptibility in the three sites even though the insecticide shared the same target site mutation responsible to the resistance to bendiocarb. These insecticides of the same target site mutation do not have the same efficacy, because the type of formulation and the dosage of the insecticides could contribute to achieving different efficacy. These different results obtained with pirimiphos-methyl and bendiocarb show the necessity or importance of combining insecticides in vector control, as proposed by the WHO in its resistance management approaches.

Clothianidin yielded 100% mortality in Gagnoa, 94.9% in Daloa and 96.6% in Abengourou. Clothianidin (2%) had a higher lethal effect on *An. gambiae* population in all study sites compared to the usual insecticides (except pirimiphos-methyl). This molecule has been shown to be effective against mosquitoes because it is new to public health. It has also been shown to have a different mode of action from those currently in use (organochlorine, pyrethroids, carbamates and organophosphates). Clothianidin is a novel neonicotinoid insecticide acting as an agonist of the nicotinic acetylcholine receptor (nAChR). This receptor is different from those of the existing recommended insecticide families [46]. To maintain its effectiveness, it should be combined or alternated with other molecules. However, it should not be overlooked that in Daloa and Abengourou, even after seven days of observation, they have still alive mosquitoes. Therefore, vectors from the study sites and indirectly from all districts of Côte d’Ivoire still need to be monitored in order to mitigate a probable resistance development to this molecule.

The 100 µg/bottle dose of chlorfenapyr induced a 100% mortality in Daloa and Gagnoa and 98% in Abengourou. These results are much better than those obtained with pyrethroids. In some cases, these studies have helped to understand and delimit the appropriate diagnostic doses for testing sensitivity to chlorfenapyr [17, 30, 47]. This molecule gives us great hope in the face of resistance developed by mosquitoes. Like clothianidin, it is also a molecule with a different mode of action to pyrethroids, and is also new to public health. Chlorfenapyr is a protoxin requiring activation, via cytochrome P450 monooxygenases, to exert its toxic effects, via uncoupling of oxidative phosphorylation [48]. This could explain the high mortality rates observed with this molecule. These results could support a resistance management plan by proposing an insecticide rotation strategy for vector control interventions and the use of new types of net incorporating either PBO or chlorfenapyr in Côte d'Ivoire.

The *kd*r-*west* mutation involving in resistance to pyrethroids insecticides was present at high frequency (0.58 to 0.72) in the three study areas. The G280S mutation was also present (0.19 to 0.29) in these areas. The high allelic frequency R in the population would indicate at molecular level a substitution of leucine by phenylalanine. This mutation, which is common in *An. gambiae* populations in West Africa, would have conferred a high level of resistance to permethrin and cross-resistance to all pyrethroids [49]. This prevents the insecticides from penetrating the entire nervous system of mosquitoes and preventing them from dying. These results highlight the resistance status observed with currently used insecticides including pyrethroids insecticides and bendiocarb. Similar results have been found by several researchers from Côte d’Ivoire and elsewhere [8, 9, 44, 50, 51]. But the *kdr-east* mutation was very low in the Abengourou (0.02) and Daloa (0.02) samples and almost absent in Gagnoa. The low proportion of this mutation in *An. gambiae* populations may be due to its recent appearance in Côte d’Ivoire. Indeed, *kdr*-*east* mutation was reported for the first time in Côte d'Ivoire in 2017 [52]. These results confirm that the search for these genes in other localities need to be intensified, as its spread could also compromise vector control efforts.

## Conclusion

This study demonstrated that the 2% clothianidin and the 100 µg/bottle of chlorfenapyr induced high mortality in natural and pyrethroid-resistant *An. gambiae s.l.* populations from Côte d'Ivoire. These results suggested increased monitoring of vector susceptibility in more localities to select the appropriate insecticides and concentrations for each district to better allocate insecticide-based vector control tools. These results could support a resistance management plan by proposing an insecticide rotation strategy for vector control interventions.

## Data Availability

The datasets generated and/or analysed during the current study are available from the corresponding author on reasonable request.

## Abbreviations

WHO: World Health Organization
KDR: Knockdown Resistance
LLINs: Long-Lasting Insecticidal Nets
PBO: Piperonyl butoxide
CDC: Centers for Disease Control and Prevention
